# Effect of PEEP and Tidal Volume on Ventilation Distribution and End-Expiratory Lung Volume: A Prospective Experimental Animal and Pilot Clinical Study

**DOI:** 10.1371/journal.pone.0072675

**Published:** 2013-08-22

**Authors:** Günther Zick, Gunnar Elke, Tobias Becher, Dirk Schädler, Sven Pulletz, Sandra Freitag-Wolf, Norbert Weiler, Inéz Frerichs

**Affiliations:** 1 University Medical Center Schleswig-Holstein, Campus Kiel, Department of Anesthesiology and Intensive Care Medicine, Kiel, Germany; 2 University Medical Center Schleswig-Holstein, Campus Kiel, Institute of Medical Informatics and Statistics, Kiel, Germany; University of Giessen Lung Center, Germany

## Abstract

**Introduction:**

Lung-protective ventilation aims at using low tidal volumes (V_T_) at optimum positive end-expiratory pressures (PEEP). Optimum PEEP should recruit atelectatic lung regions and avoid tidal recruitment and end-inspiratory overinflation. We examined the effect of V_T_ and PEEP on ventilation distribution, regional respiratory system compliance (C_RS_), and end-expiratory lung volume (EELV) in an animal model of acute lung injury (ALI) and patients with ARDS by using electrical impedance tomography (EIT) with the aim to assess tidal recruitment and overinflation.

**Methods:**

EIT examinations were performed in 10 anaesthetized pigs with normal lungs ventilated at 5 and 10 ml/kg body weight V_T_ and 5 cmH_2_O PEEP. After ALI induction, 10 ml/kg V_T_ and 10 cmH_2_O PEEP were applied. Afterwards, PEEP was set according to the pressure-volume curve. Animals were randomized to either low or high V_T_ ventilation changed after 30 minutes in a crossover design. Ventilation distribution, regional C_RS_ and changes in EELV were analyzed. The same measures were determined in five ARDS patients examined during low and high V_T_ ventilation (6 and 10 (8) ml/kg) at three PEEP levels.

**Results:**

In healthy animals, high compared to low V_T_ increased C_RS_ and ventilation in dependent lung regions implying tidal recruitment. ALI reduced C_RS_ and EELV in all regions without changing ventilation distribution. Pressure-volume curve-derived PEEP of 21±4 cmH_2_O (mean±SD) resulted in comparable increase in C_RS_ in dependent and decrease in non-dependent regions at both V_T_. This implied that tidal recruitment was avoided but end-inspiratory overinflation was present irrespective of V_T_. In patients, regional C_RS_ differences between low and high V_T_ revealed high degree of tidal recruitment and low overinflation at 3±1 cmH_2_O PEEP. Tidal recruitment decreased at 10±1 cmH_2_O and was further reduced at 15±2 cmH_2_O PEEP.

**Conclusions:**

Tidal recruitment and end-inspiratory overinflation can be assessed by EIT-based analysis of regional C_RS_.

## Introduction

It is well known that mechanical ventilation leads to lung injury. Dreyfuss and coworkers comprehensively showed the relationship between mechanical ventilation and pathologic lung tissue changes [1]. Liable factors inducing lung injury are high plateau pressures (P_plat_), high tidal volumes (V_T_) and cyclic opening and closing of alveoli (tidal recruitment) [2].

To minimize ventilator-induced lung injury the concept of lung-protective ventilation was introduced comprising the limitation of P_plat_, reduction of V_T_ and optimization of positive end-expiratory pressure (PEEP) [3]–[5]. The current recommendations advocate V_T_ of 6 ml/kg predicted body weight and P_plat_ lower than 30 cm H_2_O in patients with acute lung injury (ALI) and acute respiratory distress syndrome (ARDS). Whether this recommended V_T_ is optimal or whether the risk of ventilator-induced lung injury can be reduced by further reduction of V_T_ is still under debate. However, V_T_ lower than 6 ml/kg predicted body weight poses the risk of impaired alveolar ventilation and oxygenation [6], [7].

Proposed measures to avoid derecruitment are increased PEEP and repeated recruitment maneuvers [8]. The effectiveness of these strategies to improve oxygenation was demonstrated in multiple studies [9], [10], [11]. However, other studies revealed that these measures resulted in regional lung overinflation with subsequent lung tissue injury [8], [12], [13]. This may be one of the reasons why studies comparing different PEEP strategies were inconclusive and mostly failed to demonstrate the benefit of PEEP [5], [8], [14], [15]. Individualized identification of optimal PEEP and P_plat_ according to mechanical properties of the lung and the thorax is discussed as a potential solution to the dilemma [4], [16]. The goal should be to recruit the lung, to avoid tidal recruitment and end-inspiratory overinflation.

Electrical impedance tomography (EIT) may offer new diagnostic possibilities in the assessment of recruitment (increase in end-expiratory lung volume (EELV)), tidal recruitment (change in respiratory system compliance (C_RS_) with different V_T_) and end-inspiratory overdistension (decrease in C_RS_) in mechanically ventilated patients with ARDS. Since EIT can determine regional distribution of ventilation, changes in EELV [17]–[20], and C_RS_ [21], [22] it might be applied at the bedside in addition to the measurement of global respiratory mechanics and gas exchange. Previous studies employed EIT to identify recruitment, derecruitment and overinflation. In these studies the following strategies were mainly used: stepwise variation of PEEP [23] and the low flow inflation [24], [25] or the stepwise airway pressure increase [26].

To our knowledge, it is not predictable how distribution of ventilation, regional EELV, and regional C_RS_ are affected by concomitant changes in V_T_ and PEEP in patients with severe ARDS. Thus, the goal of this experimental study was to perform EIT measurements during ventilation with low and high V_T_ at a preset PEEP to detect tidal recruitment. We hypothesized that low PEEP would lead to tidal recruitment whereas high PEEP, especially in combination with high V_T_, would lead to overinflation of at least some parts of the lung. The model and the hypothesis on which the model is based are described in more detail in Text S1 and Figure S1. We additionally checked whether this EIT-based analysis revealed tidal recruitment and end-inspiratory overinflation in pilot examinations in critically ill patients with mild and moderate ARDS.

## Materials and Methods

### Experimental study

The experimental study was performed on ten anesthetized supine pigs of both sexes (Deutsches Landschwein, Institute of Animal Breeding and Husbandry, Christian Albrechts University, Kiel, Germany) with a body weight (BW) of 50±5 kg (mean±SD). It was carried out in strict accordance with the guidelines on animal experimentation. The protocol was approved by the Committee for Animal Care of the Christian-Albrechts University, Kiel, Germany (Permit Number: V 742-72241.121-39 (80-10/03)). All surgery was performed under anesthesia with propofol and sufentanile and all efforts were made to minimize suffering.

### Animal preparation

After sedation with azaperon (8 mg/kg BW) and atropine (0.1 mg/kg) general anesthesia was started with ketamine (5 mg/kg BW), sufentanile (0.2 µg/kg BW) and propofol (1 mg/kg BW). Anesthesia was continued with intravenous infusion of propofol (6 to 12 mg/kg BW per hour) and sufentanile (10 µg/kg BW per hour). Vecuronium bromide (0.1 mg/kg BW) was administered for muscle paralysis. An infusion of lactated Ringeŕs solution was given at a rate of 20 ml/kg BW per hour. If hypovolemia was suspected additional hydroxyethyl starch and Ringeŕs solution were given. Norepinephrine was infused to maintain the mean arterial pressure above 70 mm Hg.

### Mechanical ventilation and induction of ALI

Throughout the experiment, the animals were ventilated in a volume-controlled mode (Avea, CareFusion, Höchberg, Germany) at 20 breaths/min with the ratio of inspiration and expiration times (I∶E) of 1∶1.5. ALI was induced by repeated bronchoalveolar lavage with 1.5 L of warm saline solution until arterial partial pressure of oxygen (P_a_o_2_) remained stable below 100 torr at an inspired oxygen fraction (F_I_o_2_) of 1.0 and PEEP of 10 cm H_2_O for 30 min. The animals were ventilated with V_T_ of 5 and 10 ml/kg BW both before and after ALI induction. These are referred to as low and high V_T_ throughout the text.

A constant low-flow (2.5 l/min) inflation (pressure-volume (PV)) maneuver with an inspiratory volume limit of 1.5 l was performed starting at zero end-expiratory pressure. Afterwards, positive end-expiratory pressure (PEEP) was set 2 cm H_2_O above the lower inflection point (LIP) identified in the PV curve. An additional image file shows the PV curve obtained from a representative animal (Figure S2).

### Interventional lung assist device

A pumpless extracorporeal interventional lung assist device (ILA, Novalung, Hechingen, Germany) was applied in all animals allowing control of arterial partial pressure of carbon dioxide (P_a_co_2_) independent of the ventilator pattern [27], [28]. A 13 Fr cannula was inserted into the iliac artery with ultrasound guidance and a 15 Fr cannula into the iliac vein using Seldingeŕs technique. The ILA device was prefilled with saline solution and connected to both cannulae. 5000 units of heparin were given after the instrumentation was completed. ILA was only used after induction of ALI during low V_T_ ventilation. Oxygen flow was set to 10 l/min to achieve a P_a_co_2_ of 40 mmHg.

### Electrical Impedance Tomography

EIT measurements were performed with the Goe-MF II system (CareFusion, Höchberg, Germany) using a set of 16 electrodes (Blue Sensor BR-50-K, Ambu, Ølstykke, Denmark) placed on the thoracic circumference at the fifth-sixth intercostal space. EIT data were acquired with a scan rate of 25 Hz. EIT images were generated using the filtered back-projection algorithm [29]. The data were filtered using a digital low-pass filter with a cut-off frequency of 1 Hz to eliminate small impedance changes synchronous with the heart beat.

### Experimental protocol

**Figure 1:**
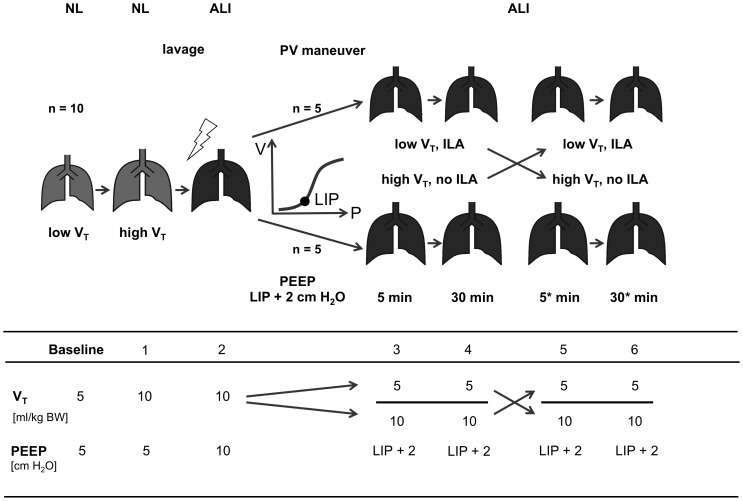
Study flowchart. Ten animals were studied during volume-controlled ventilation during ventilation with 5 ml/kg (baseline) and 10 ml/kg tidal volume (V_T_) (measurement time point 1) in the normal lung (NL) as well as after induction of acute lung injury (ALI) (time point 2) at inspired fractions of oxygen of 0.5 and 1.0, respectively. Then a constant low-flow inflation maneuver (pressure-volume (PV) maneuver) was performed and positive end-expiratory pressure (PEEP) was set 2 cm H_2_O above the lower inflection point (LIP) identified in the PV curve. Using a crossover design, further measurements were performed 5 and 30 min after ventilation with low V_T_ and active interventional lung assist (ILA) and after another 5 and 30 min with high V_T_ and inactive ILA (no ILA) (time points 3–6). Five animals were randomly ventilated in the reversed chronological order. Ventilator settings of V_T_ and PEEP at each measurement time point are shown in the lower part of the Figure. *, time elapsed after the change in ventilator and ILA settings.

A flowchart of the experimental protocol is provided in Figure 1. EIT scanning was performed during baseline conditions and at six subsequent measurement time points as described below:

- Baseline: Pigs were ventilated with F_I_o_2_ of 0.5, V_T_ of 5 ml/kg BW and PEEP of 5 cm H_2_O. ILA was inactive.
- Time point 1: V_T_ was set to 10 ml/kg BW while other settings remained unchanged.
- Time point 2: After induction of ALI, F_I_o_2_ was set to 1.0,V_T_ 10 ml/kg BW, PEEP 10 cm H_2_O and ILA inactive.

Animals were then allocated to either ventilation with low V_T_ and active ILA or to ventilation with high V_T_ and inactive ILA in randomized order (5 animals in each randomization arm). After 30 minutes, the applied V_T_/ILA pattern was changed in a crossover design where each animal served as its own control.

- Time points 3 and 5: 5 minutes after ventilation with the set V_T_/ILA pattern.
- Time points 4 and 6: 30 minutes after ventilation with the set V_T_/ILA pattern.

At baseline and at each measurement time point, heart rate, mean arterial pressure, inspiratory peak pressure (P_insp_), plateau pressure (P_plat_) and end-expiratory partial pressure of CO_2_ (Pco_2_) were determined and EIT data acquired during 60 seconds. (To account for the crossover design, the data obtained at identical V_T_/ILA settings were combined. This resulted in the merged time points 5/3 and 6/4 for low V_T_ and 3/5 and 4/6 for high V_T_ ventilation).

Blood gases were measured at time point 1, after ALI induction (i.e. time point 2), immediately after the pressure-volume maneuver was performed (during ventilation with high V_T_ and PEEP set 2 cmH_2_O above LIP) and at the end of the experiment to check for the stability of the ALI model.

### EIT data analysis

**Figure 2:**
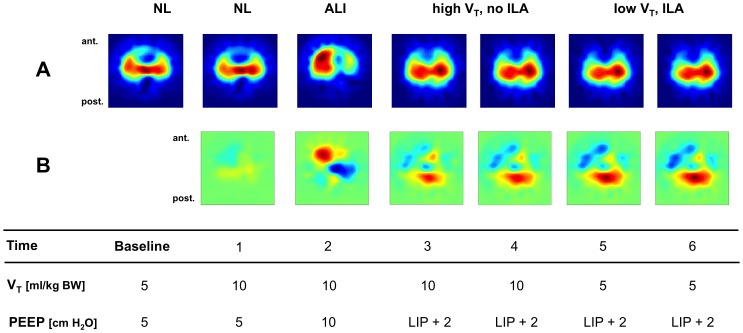
Regional ventilation distribution. Examples of functional EIT scans of regional lung ventilation in animal 2 during all measurement time points (see Figure 1 for explanation). The orientation of the scans is indicated (ant., anterior; post., posterior). Panel A: Ventilated areas within the chest cross-section exhibit higher values of relative impedance change and are shown in red tones. In normal lungs (NL), symmetrical ventilation distribution between the right and left lung regions was found. With induction of acute lung injury (ALI), higher ventilation in the right lung region with pronounced ventilation in its ventral part and reduced left lung ventilation especially in its dorsal part were found. After PEEP was set 2 cm H_2_O above the lower inflection point (LIP) according to the pressure-volume curve, a shift in ventilation toward the dependent (dorsal) lung regions was observed. No obvious difference between ventilation with 10 ml/kg V_T_ and inactive interventional lung assist (ILA) (high V_T_, no ILA) and ventilation with 5 ml/kg V_T_ and active ILA (low V_T_, ILA) was detected. Panel B: Ventilation difference scans of the same animal. Red color indicates increase in regional ventilation, blue color shows the decrease in ventilation compared with baseline.

Functional EIT scans were generated from each measurement using an established approach [19]. They showed the distribution of regional V_T_ in the chest cross-section by calculating the tidal amplitudes of relative impedance change in 912 image pixels (Figure 2).

Ventrodorsal profiles of fractional V_T_ in 32 layers were generated from the functional ventilation scans and the geometrical centers of ventilation were calculated in relation to the ventrodorsal chest diameter as previously described [30]–[32].

**Figure 3:**
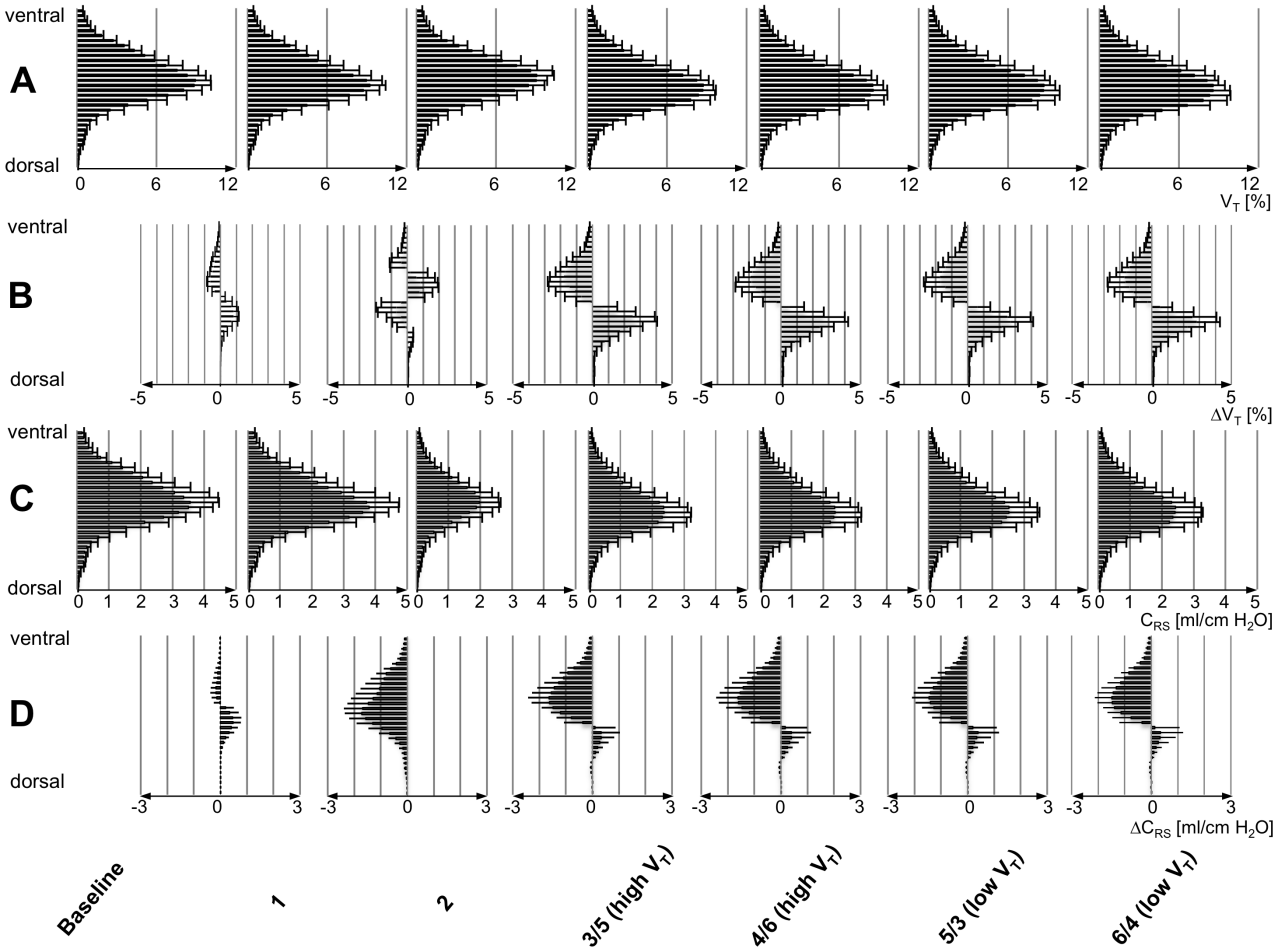
Regional ventilation and respiratory system compliance. Ventrodorsal profiles of regional tidal volume (V_T_) (A), regional respiratory system compliance (C_RS_) (C) and differences in regional V_T_ (ΔV_T_) (B) and regional C_RS_ (ΔC_RS_) (D) with respect to baseline (mean values ±SD in 10 animals). Panel A (V_T_ [%]) shows the relative distribution of V_T_ in 32 horizontal layers in% of overall V_T_ in the chest cross-section. Panel B (ΔV_T_ [%]) indicates the respective differences in regional V_T_ compared with baseline. It shows a shift in ventilation toward the dorsal regions already with higher V_T_ at time point 1 in normal lung and more pronounced shifts at points 3 through 6 with a PEEP set 2 cm H_2_O above the lower inflection point in acute lung injury (ALI). Panel C (C_RS_ [ml/kg H_2_O]) shows regional C_RS_ in the same 32 layers with the respective differences to baseline provided in panel D (ΔC_RS_ [ml/kg H_2_O]). The differences in regional C_RS_ indicate slightly lower and higher C_RS_ in the ventral and dorsal regions at time point 1. Significantly lower values were found in layers 7 to 12 and significantly higher ones in layers 16 to 25 (layers counting from 1 to 32 in the ventrodorsal direction). ALI (time point 2) resulted in significant decrease in C_RS_ in all layers. A small increase in C_RS_ in the dorsal regions (significant in layers 23 and 24) with the decreased C_RS_ in the ventral regions (significant in layers 1 to 17 and 27 and 28) at time point 3/5. At all other three time points, the findings were comparable. High V_T_, ventilation with 10 ml/kg BW, low V_T_, ventilation with 5 ml/kg BW.

To compare and display changes in ventilation distribution between the baseline and individual measurement time points, EIT ventilation difference images [33] and regional ventilation difference profiles were generated (Figures 2 and 3). Changes in EELV between individual time points and baseline were analyzed by calculating the differences between the minimum (end-expiratory) values of ventilation-related relative impedance change.

Finally, global C_RS_ was calculated as V_T_/(P_plat_−PEEP) and regional C_RS_ determined in each of the 32 layers as ((fraction of V_T_)·V_T_)/(P_plat_−PEEP). Corresponding to the ventilation profiles described above, regional C_RS_ and C_RS_ difference profiles were generated (Figure 3).

### Pilot patient study

The study was approved by the Ethics committee of the Christian-Albrechts University, Kiel, Germany (“Bestimmung der globalen und regionalen Atemmechanik bei unterstützter Spontanatmung” Permit Number: A 125/12). Written informed consent was obtained from each patient or their legal representative, respectively.

We included five adult patients (age 74±6 years, height 174±5 cm, weight 77±15 kg) with mild and moderate ARDS according to the Berlin definition [34] treated in our surgical intensive care unit. ARDS resulted from severe sepsis (n = 4) and pneumonia (n = 1). Anaesthesia was performed with sufentanile and propofol and if required for an intervention or intubation muscle paralysis was induced with rocuronium. Patients were ventilated with Evita XL (Dräger Medical AG & Co., Lübeck, Germany) in the pressure-controlled mode. During the study period volume-controlled mode was used. A low-flow (4 L/min) PV maneuver was started at PEEP of 0 cm H_2_O with an inspiratory volume limit of 2 L and a pressure limit of 35 cm H_2_O. Afterwards, PEEP was set at three different levels according to the LIP. We started with a PEEP of LIP+2 cm H_2_O followed by LIP−5 cm H_2_O and LIP+7 cm H_2_O. At each PEEP level, patients were first ventilated with low V_T_ (6 ml/kg BW) followed by high V_T_ (10 ml/kg BW) for 5 minutes. The lowest PEEP was second in order to avoid unnecessary long derecruitment and followed by the highest PEEP. We a priori decided not to exceed a pressure limit of 40 cm H_2_O at this PEEP level and, therefore, we reduced V_T_ to 8 ml/kg BW.

EIT examinations and data analyses were performed exactly as described above in sections on the experimental study. (The only difference was that we used the L-00-S electrodes (Ambu, Ølstykke, Denmark) in patients.) We calculated C_RS_ at each PEEP level and each V_T_ and the C_RS_ differences between high and low V_T_ at each PEEP level.

### Statistical analysis

Since normal distribution assumption was not violated (Shapiro-Wilk-test), data are presented as means±SD and analyzed parametrically with paired t test or repeated-measures ANOVA as appropriate. The pilot patient data were analyzed using descriptive statistics as means±SD.

The statistical analysis was conducted using SPSS version 17.0 (SPSS Inc., Chicago, IL, USA). All statistical tests were two-sided and the level of significance was set at 5%.

## Results

### Experimental study

#### Hemodynamics and global respiratory system mechanics

All animals studied were included in the final analyses. Hemodynamic and respiratory data are summarized in Table 1. Pco_2_ increased to about 60 torr during ventilation with high V_T_ and inactive ILA whereas ventilation with low V_T_ and active ILA resulted in normocapnia. Increasing V_T_ from 5 to 10 ml/kg BW did not significantly change global C_RS_ in the healthy lungs. ALI reduced C_RS_ markedly to about 50% of baseline values despite PEEP increase to 10 cm H_2_O. Application of PEEP according to LIP restored C_RS_ only partially.

**Table 1:** Respiratory and hemodynamic data.

Time point	Baseline	1	2	3/5	4/6	5/3	6/4
				(high V_T_)	(high V_T_)	(low V_T_)	(low V_T_)
**F_I_o_2_**	0.5	0.5	1	1	1	1	1
**V_T_ [ml]**	252±35	494±59	487±67	466±95	466±95	255±31	255±31
**PIP [cm H_2_O]**	13±2	20±2†	35±3§	42±6§	41±6§	31±4†	31±4†
**Plateau pressure**	12±2	17±3†	32±4§	36±9§	36±8§	27±7†	28±7†
**[cm H_2_O]**							
**PEEP [cm H_2_O]**	5	5	10	21±4	21±4	21±4	21±4
**C_RS_ [ml/cm H_2_O]**	40±10	42±10	22±7§	27±8§	28±8§	29±9* ^$^	28±8* ^&^
**Pco_2_ [torr]**	46±10	39±9*	51±14	58±19††	57±21††	40±10‡	42±12*
**MAP [mm Hg]**	83±18	77±12	72±15	76±13	71±9	71±7	67±5*
**HR [1/min]**	95±14	99±13	132±16‡‡	131±21‡‡	132±17‡‡	125±13	132±16

Data are shown as mean values ± standard deviation. V_T_: tidal volume, F_I_o_2_: fraction of inspired oxygen, PEEP: positive end-expiratory pressure, PIP: peak inspiratory pressure, C_RS_: respiratory system compliance, Pco_2_: end-expiratory partial pressure of carbon dioxide, MAP: mean arterial pressure, HR: heart rate. Due to the crossover design data obtained at identical V_T_/ILA settings were combined resulting in the merged time points 5/3 and 6/4 for low V_T_ and 3/5 and 4/6 for high V_T_ ventilation.

*: vs. baseline (P<0.05).

‡ : vs. baseline (P<0.01).

† : vs. baseline (P<0.0001).

†† : vs. time point 1 (P<0.05).

‡‡ : vs. time point 1 (P<0.001).

§ : vs. time point 1 (P<0.0001).

$: vs. time point 2 (P<0.01).

&: vs. time point 2 (P<0.05).

#### Regional distribution of ventilation and respiratory system compliance

**Figure 4:**
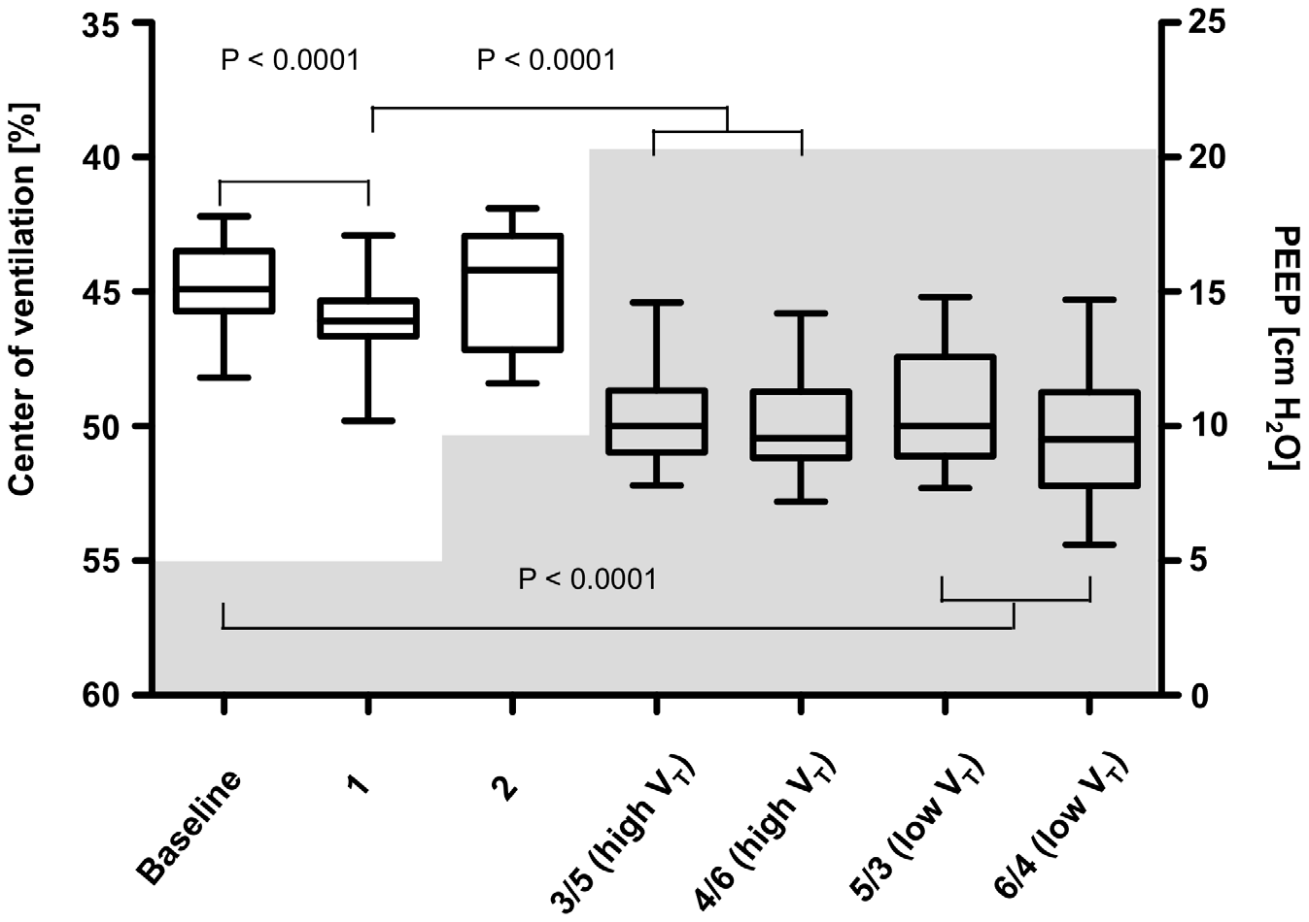
Center of ventilation. Ventilation distribution during individual measurement time points represented by the geometrical center of ventilation. The center of ventilation is given in percent of the anteroposterior chest diameter. Values above 50 indicate a location in the dorsal half of the chest cross-section. The median, the 25th and the 75th percentile, minimum and maximum values of ten animals are shown. The gray areas in the diagram show the positive end-expiratory pressure (PEEP) values during the individual measurement time points. Significant differences between corresponding high V_T_ and low V_T_ are indicated. Every group with ALI and high PEEP is significantly different from normal lung and ALI with PEEP 10 cm H_2_O (Time point 2). Left Y axis: center of ventilation, right Y axis: PEEP. High V_T_, ventilation with 10 ml/kg BW, low V_T_, ventilation with 5 ml/kg BW.

Increasing V_T_ in the healthy lung from 5 to 10 ml/kg BW led to a small but significant redistribution of ventilation in favor of the dependent lung regions. The geometrical center of ventilation moved slightly but significantly downwards (Figure 4). This went along with an increase in regional C_RS_ in the dependent parts of the lung (Figure 3).

In ALI, a homogenous reduction in C_RS_ throughout the lung was observed during ventilation with high V_T_ despite increased PEEP of 10 cmH_2_O (Figure 4). Distribution of ventilation was not different compared to the healthy lung (Figure 4). Increasing PEEP to 2 cm H_2_O above the LIP of the pressure-volume curve restored regional C_RS_ to initial values in the dependent lung regions and reduced it in the non-dependent ones. Accordingly, distribution of ventilation was directed more toward the dependent lung regions as reflected by the downward shift in geometrical centers of ventilation. There was no difference in ventilation distribution and regional C_RS_ between ventilation with 5 or 10 ml/kg BW at the four measurement time points with PEEP increased to 2 cm H_2_O above LIP (Figures 3 and 4).

#### End-expiratory lung volume

**Figure 5:**
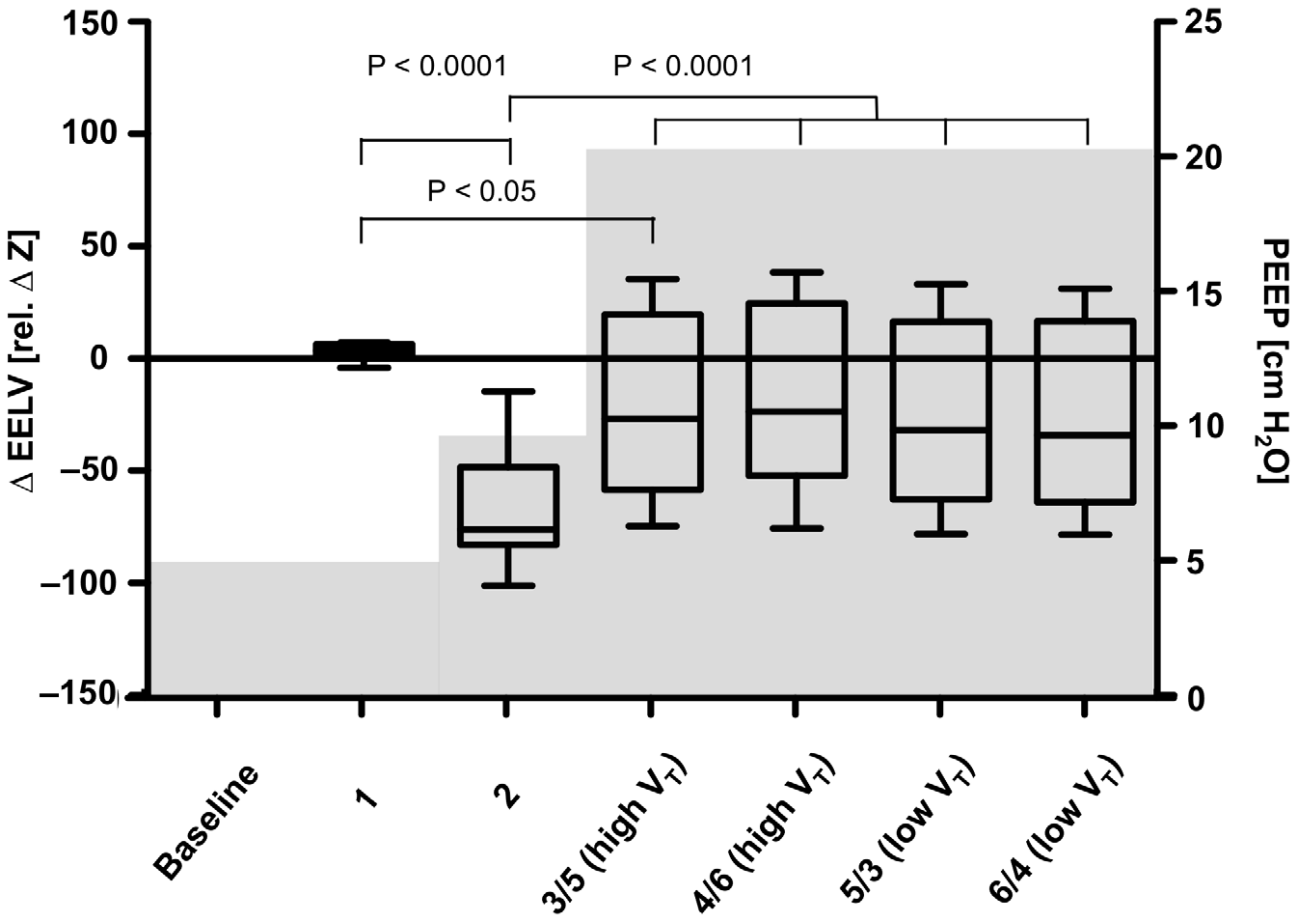
End-expiratory lung volume. Changes in end-expiratory lung volume (ΔEELV) at individual measurement time points in comparison to baseline. The median, the 25th and the 75th percentile, minimum and maximum values of ten animals are shown. The gray areas in the diagram show the positive end-expiratory pressure (PEEP) values during the individual time points. Significant differences between the measurements are indicated. Left Y axis: ΔEELV, right Y axis: PEEP. High V_T_, ventilation with 10 ml/kg BW, low V_T_, ventilation with 5 ml/kg BW.

Regardless of the applied V_T_, EELV did not change in the healthy lung (Figure 5). ALI led to a pronounced loss in EELV despite PEEP of 10 cm H_2_O. This volume loss could only partially be regained when increased PEEP was set according to the pressure-volume curve with high variability among the animals (Figure 5).

#### Stability of the model

There were no significant differences in gas exchange between the initial measurement after induction of ALI and the final measurements at the end of the experimental protocol (Table 2).

**Table 2:** Gas exchange data.

Time point	Normal lung	ALI	PV maneuver	End of experiment
	(time point 1)	(time point 2)		
**F_I_o_2_**	0.5	1	1	1
**P_a_o_2_ [torr]**	234±40†	120±27** †	420±110	133±27†
**P_a_o_2_/F_I_o_2_ [torr]**	468±80	120±27	420±110	133±27
**P_a_co_2_ [torr]**	47±10	58±11*	58±15	60±18*

Data are shown as mean values ± standard deviation. ALI: acute lung injury, F_I_o_2_: arterial oxygen saturation, P_a_o_2_: arterial partial pressure of oxygen, P_a_co_2_: arterial partial pressure of carbon dioxide, P: pressure, V: volume.

At the time point ‘PV maneuver’, data was obtained immediately after the low-flow inflation maneuver during ventilation with high V_T_ and with PEEP set 2 cmH_2_O above the lower inflection point.

*: vs. time point 1 (P<0.05).

**: vs. time point 1 (P<0.001).

† : vs. PV maneuver (P<0.0001).

### Pilot patient study

**Figure 6:**
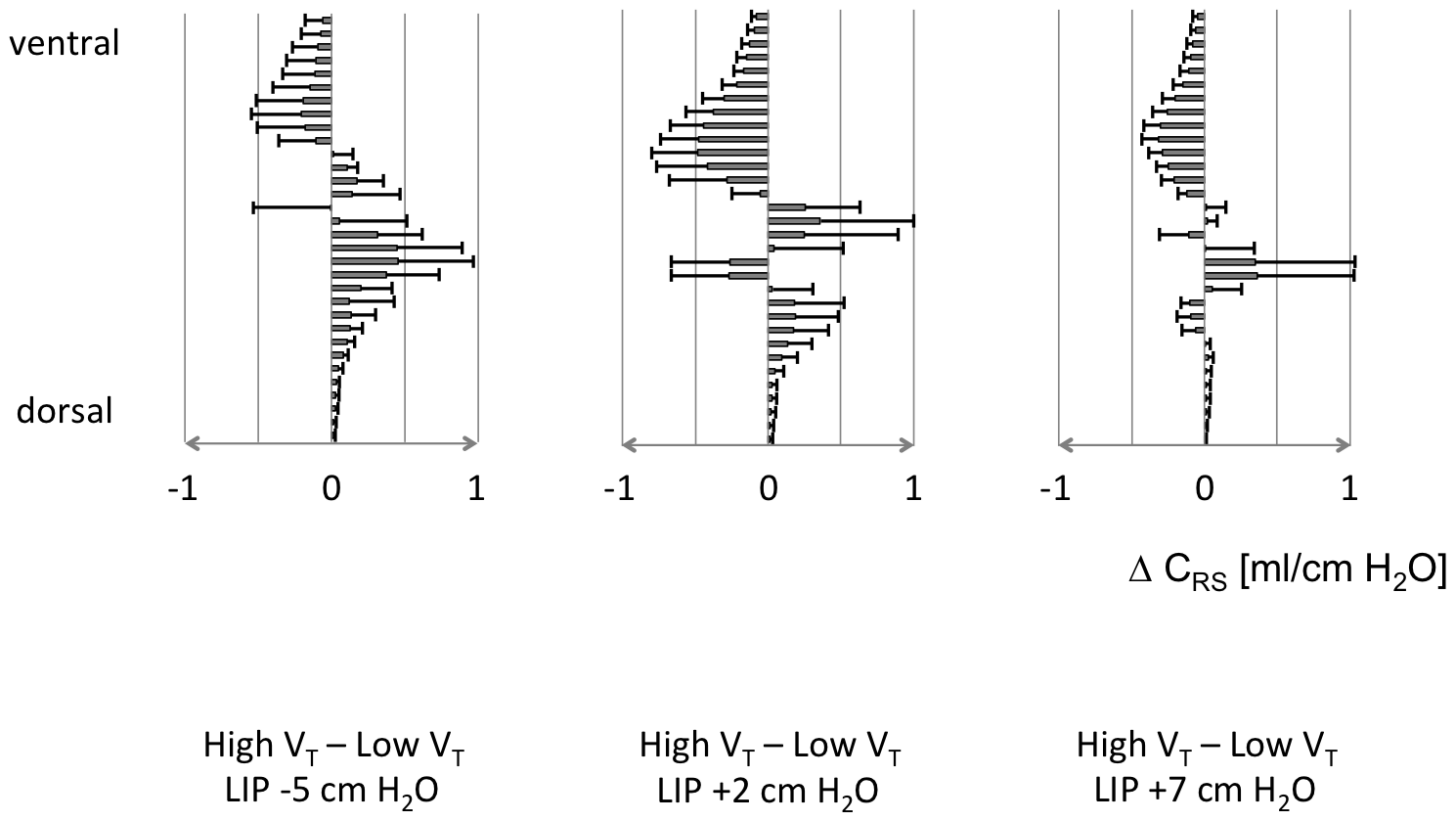
Regional differences in respiratory system compliance. Differences in regional respiratory system compliance (ΔC_RS_) in 32 regions of interest in the chest cross-section in five patients with ARDS between ventilation at high and low tidal volume (V_T_) at three levels of positive end-expiratory pressure set according to the lower inflection point (LIP) of the quasistatic pressure-volume curve. ΔC_RS_ are presented as ventrodorsal profiles. Values are means ±SD.

Respiratory and hemodynamic data are given in Table 3. LIP was identified at 8±2 cm H_2_O. Regional C_RS_ differences at the respective PEEP levels are shown in Figure 6. At the lowest PEEP of 3±1 cm H_2_O we found a pronounced increase in C_RS_ in the dorsal regions and a small decrease in the ventral regions with high V_T_. At PEEP of 10±1 cm H_2_O, high V_T_ led to a smaller increase in C_RS_ in the dorsal regions and more pronounced reduction in the ventral parts than at the lowest PEEP. At PEEP of 15±2 cm H_2_O, the C_RS_ difference between high and low V_T_ was even smaller.

**Table 3:** Respiratory and hemodynamic data of the studied patients.

Time point	Baseline	Low V_T_	High V_T_	Low V_T_	High V_T_	Low V_T_	High V_T_
		LIP−5	LIP−5	LIP+2	LIP+2	LIP+7	LIP+7
**P_a_o_2_/F_I_o_2_ [torr]**	213±63		183±52		244±62		277±61
**V_T_ [ml]**		418±40	705±68	418±41	709±61	419±34	554±46
**PIP [cm H_2_O]**		16±1	26±2	24±2	34±4	30±2	37±2
**Plateau pressure**		13±1	19±1	20±2	28±3	28±1	33±3
**[cm H_2_O]**							
**PEEP [cm H_2_O]**		3±1	3±1	10±1	10±1	15±2	16±2*
**C_RS_ [ml/cm H_2_O]**		43±7	44±8	42±7	39±9	36±8	36±10
**P_a_o_2_ [torr]**	99±23		85±13		114±21		130±30
**P_a_co_2_ [torr]**	51±12		59±12		59±13		64±10
**HR [1/min]**	90±20	85±15	79±18	84±15	80±17	84±16	77±11

Data are shown as mean values ± standard deviation. V_T_: tidal volume, F_I_o_2_: fraction of inspired oxygen, PEEP: positive end-expiratory pressure, PIP, peak inspiratory pressure, C_RS_: respiratory system compliance, P_a_co_2_: end-expiratory partial pressure of carbon dioxide, HR: heart rate.

*The measurement at LIP+7 and high V_T_ was not conducted in patient 1 due to excess of peak inspiratory pressure limit of 40 cm H_2_O (see Methods for further details).

## Discussion

Our study examined regional ventilation, C_RS_ and EELV using EIT at different V_T_ and PEEP in lung healthy animals and after induction of ALI. The protocol was designed to reflect clinical decision-making regarding the choice of PEEP and V_T_ in ARDS patients. It tested the hypothesis that a variation of tidal volume at a preset PEEP could be used to assess tidal recruitment and therefore guide the choice of adequate (optimum) PEEP. We subsequently applied this EIT-based approach developed in the experimental study in a small pilot study in mechanically ventilated patients.

In the experimental study, we found that an increase in V_T_ from 5 to 10 ml/kg BW led to an increase in regional C_RS_ and fractional ventilation in the dependent parts of the healthy lung. This is in accordance with our hypothesis that the application of different V_T_ at a distinct PEEP can be used in combination with EIT to assess the recruitment potential of the lung. The increase in C_RS_ in the dependent parts of the lung with high V_T_ is hereby interpreted as an increase in ventilated volume in these parts of the lung (Figure S1). With identical mechanical properties, an increase in ventilated volume leads to an increase in compliance. ALI induced a profound reduction in EELV accompanied by low global and regional C_RS_ despite the application of a PEEP of 10 cm H_2_O. Surprisingly, the distribution of ventilation was similar to the healthy lung. The subsequent further PEEP increase according to the identified LIP of the pressure-volume curves restored C_RS_ to near baseline values in the dependent lung regions. However, C_RS_ in the ventral regions of the lung was markedly decreased implying overinflation. The fact that there was no difference in regional C_RS_ and distribution of ventilation between V_T_ of 5 and 10 ml/kg BW suggests that no tidal recruitment occurred. The fact that EELV did not reach the level observed in the normal lung even with the high PEEP has to be interpreted with caution, since the induction of ALI may have changed the electrical conductivity of the lung tissue [35].

### Recruitment and derecruitment

Our finding of dorsally directed shift in ventilation and increase in regional C_RS_ in the dependent lung regions at a PEEP of 5 cm H_2_O and high V_T_ in normal lungs is in accordance with previous findings. Sinclair et al. who examined the effect of different PEEP (0 and 8 cm H_2_O) and V_T_ (6 and 12 ml/kg) on cyclic airway collapse and recruitment using aerosolized fluorescent microspheres in a rabbit model revealed that cyclic tidal recruitment occurred with low PEEP in the healthy lung [36]. Improved ventilation in the dorsocaudal lung regions with high PEEP in that study was attributed to local recruitment with a postulated increase in regional C_RS_.

In our study, a PEEP of 10 cm H_2_O was not sufficiently high to prevent derecruitment after ALI induction as reflected by overall decrease in regional C_RS_. This derecruitment could be reversed after the pressure-volume maneuver and subsequent application of higher PEEP of about 21 cm H_2_O. At the same time, however, regional C_RS_ fell in the non-dependent regions when compared with baseline. Thus, although PEEP had the beneficial effect of recruiting the lung in the dependent regions and thereby avoiding tidal recruitment it also lead to regional overdistension. This phenomenon was also identified by Grasso et al. in three different pig models of ALI, including the lavage model, where overinflation was present in the baby lung despite recruited lung areas [6]. The concomitant existence of lung regions exhibiting recruitment and overinflation was also determined in patients with ALI [37], which renders the selection of adequate PEEP so difficult in individual patients.

### End-expiratory lung volume

In our study, ALI led to a pronounced reduction in EELV, which could not be offset by PEEP of 10 cm H_2_O. By further increasing PEEP according to the pressure-volume curve, EELV increased.

Several studies have proven the ability of EIT to detect PEEP-dependent changes in EELV by analysis of end-expiratory impedance values [19], [38]–[40] as used in our analysis. The fall in end-expiratory impedance values associated with ALI development has previously been described, although in an oleic acid ALI model [33]. Previous EIT studies using either two [41] or three electrode planes [19] demonstrated that EIT-based evaluation of EELV requires a cautious selection of the electrode plane. Therefore, we chose the midthoracic plane for our EIT measurements. With this approach, most of the lung tissue was included in the analysis because the examined chest slice was approximately 10 to 15 cm thick [42].

### Ventilation distribution

To characterize regional ventilation distribution by EIT, we have generated ventrodorsal ventilation profiles derived from the functional EIT scans and calculated the centers of ventilation [30], [32], [43]. This is a relatively simple but sensitive procedure that was previously applied to determine the effects of ventilation mode, PEEP, recruitment maneuvers or surfactant administration on ventilation distribution [30], [31], [43].

The redistribution of ventilation occurring between the individual measurement time points resulted in shifts of the centers of ventilation in the ventrodorsal direction. The dorsal shift identified in the animals ventilated with high V_T_ compared with low V_T_ before ALI implied tidal recruitment in the dependent lung regions. The centers of ventilation exhibited the dorsal most locations during ventilation with high PEEP set according to the pressure-volume curve after ALI. This was consistent with reduced ventilation in the non-dependent regions caused by overinflation accompanied by an increase in ventilation in the dependent regions caused by recruitment.

To better visualize the changes in regional ventilation induced by the study interventions (i.e., ALI, PEEP and V_T_ changes), we also calculated ventrodorsal profiles showing the differences in regional V_T_ at individual time points in comparison with baseline. These profiles highlighted the ventilation changes identified by the centers of ventilation by showing the respective changes in 32 chest layers.

### Regional respiratory system compliance

Although the topographical distributions of regional V_T_ and C_RS_ have to be identical by virtue of the underlying calculation of regional C_RS_, the absolute values of regional C_RS_ reflect the changing respiratory system mechanics in the course of the experiment. This was detected especially after the induction of ALI (measurement time point 2) where a dramatic loss in regional C_RS_ was observed in all analyzed lung layers, whereas regional tidal volumes remained fairly unchanged (Figure 3).

The profiles of differences in regional C_RS_ detected the changing C_RS_ when compared with baseline: After the induction of ALI during ventilation at 10 cm H_2_O of PEEP (time point 2), a marked decrease in C_RS_ was found. Higher regional C_RS_ in the dependent lung regions at high PEEP in the later phase after ALI could be attributed to recruitment of lung tissue as shown by Sinclair et al. [36]. The simultaneous decrease in regional C_RS_ in the ventral, non-dependent regions reflected regional overinflation. These results indicate that the distribution of regional C_RS_ and regional differences in C_RS_ are crucial for the interpretation of the PEEP and V_T_ effects and that the threshold PEEP, where tidal recruitment begins or ceases, might be the optimal PEEP to achieve best possible recruitment and minimal overinflation.

The importance of regional C_RS_ has also been highlighted by two recent EIT studies. Bikker et al. calculated C_RS_ in four horizontal chest layers at 15, 10, 5 and 0 cm H_2_O of PEEP during a decremental PEEP trial [41]. They found that high PEEP led to an increase in regional C_RS_ in the dependent part of the lung indicating recruitment but also to a decrease in C_RS_ in the non-dependent part suggesting overinflation. Dargaville et al. examined regional C_RS_ in three horizontal layers of the lungs during an incremental/decremental PEEP trial using a total of 11 PEEP steps [44]. Regional recruitment, derecruitment and overinflation could be detected and the PEEP value identified at which the most homogeneous C_RS_ distribution was achieved during the deflation limb of the maneuver. Our results show that a change in regional C_RS_ with different V_T_ can be used to determine recruitment potential, implying that tidal recruitment occurs and the choice of a higher PEEP could be advantageous.

### Interventional lung assist

Based on former studies [28], [45], we presumed that ventilation with low V_T_ of 5 ml/kg BW would lead to CO_2_ accumulation in our animal model of ALI which would not allow us to maintain the ventilatory pattern constant. Since we focused on the measurement of lung mechanics we considered it essential to keep the pattern constant throughout the whole experiment. The technique of interventional lung assist is easily available in our animal laboratory, therefore, we used it for CO_2_ removal in the phases of ventilation with low V_T_. When we designed the experimental protocol, we expected from our previous experience that the ventilation with high V_T_ during ALI would result in a P_a_co_2_ of about 40 mmHg. During the experiments we saw it was slightly higher, nevertheless, we decided to adhere to our original protocol.

### Pilot patient data

In the animal experimental phase we could not identify any significant differences in C_RS_ between the low and high V_T_ after ALI because the lungs were already maximally inflated by the applied high PEEP. Therefore, we could not test our EIT-based approach of identifying tidal recruitment and end-inspiratory overinflation in the injured lungs of the studied animals. However, our pilot patient EIT data acquired at three PEEP levels, allowed us to apply this analysis. At the lowest PEEP, tidal recruitment in the dependent regions could clearly be identified. At the two higher PEEP values, progressive reduction in tidal recruitment was seen. End-inspiratory overinflation in the non-dependent regions was present already at the PEEP level of 2 cm H_2_O above LIP. At the highest PEEP, regional overdistention at the higher of the two V_T_ values was blunted by the already present PEEP-induced overinflation. We postulate that the individual optimum PEEP could be derived from similar EIT examinations at the bedside in the future: the variation of V_T_ at different PEEP levels could identify the settings with minimum tidal recruitment and minimum overinflation.

### Limitations

1. We chose a lavage model of ALI well aware of the fact that it does not closely reflect the clinical situation because it is recruitable with PEEP and high V_T_ [46]. However, this was a desired feature of the model in our study in order to evaluate the ability of EIT to detect V_T_-dependent tidal recruitment. We could show that our ALI model was stable during the experiment and did not exhibit spontaneous recruitment in the course of time.
2. We limited the experiment to the crucial measurement time points to exclude the influence of time and, therefore, we applied only the high but not the low V_T_ after ALI induction with a PEEP of 10 cm H_2_O. We did not expect lacking tidal recruitment during ventilation at high PEEP set according to the pressure-volume curve, otherwise, we would have studied both V_T_ at the lower PEEP of 10 cm H_2_O. Since the data analysis was performed offline it was too late to change the protocol. However, our pilot patient data acquired at lower PEEP values than in animal experiments could show that tidal recruitment could be reliably assessed by EIT-derived regional C_RS_.
3. EIT measurements were not compared with another established radiological imaging modality like computed tomography. This might be regarded as a limitation, however, the feasibility of EIT to assess regional ventilation has been previously validated with multiple standard imaging techniques [47]–[49].
4. EIT does not measure absolute lung volumes and thus we were only able to report relative changes in EELV. The validity of using relative instead of absolute lung volumes was previously confirmed by using the nitrogen washout technique [40].

### Conclusions

1. With a PEEP of 5 cm H_2_O, tidal recruitment was determined by EIT in the normal lung implying recruitment potential at this PEEP value.
2. PEEP set according to the pressure-volume curve at 2 cm H_2_O above LIP proved to be too high in the experimentally injured lung since no tidal recruitment was detected but pronounced regional overinflation was present in the non-dependent lung regions.
3. Regional tidal recruitment and end-inspiratory overinflation was identified in patients with ARDS with EIT by calculation of regional C_RS_ differences from measurements acquired at different V_T_ and PEEP.
4. Concomitant analysis of regional V_T_, EELV and C_RS_ using EIT holds substantial potential to titrate lung protective ventilation by facilitating choice of adequate PEEP to avoid tidal recruitment and adequate V_T_ to prevent overdistension.

## Supporting Information

Figure S1 Explanation of the model. Schematic presentation of postulated changes in regional lung ventilation and regional respiratory system mechanics during different phases of the study protocol. Each large circle symbolizes ventilated lung volume. The small blue and large red circles represent normally aerated and overdistended lung regions, respectively. The oval dark grey symbols indicate atelectatic lung regions. The transparent grey bars show schematically two of the 32 regions of interest (ROI) used in our EIT analysis. (The sizes of these representative ROIs were enlarged to enable better visual perception.) The effect of an intervention is displayed from left to right showing the compliance change in the pressure (P)-volume (V) coordinates in the respective ROI and the assumed differences in regional compliance (ΔC) in the whole lung. Upper panel: An increase in tidal volume (V_T_) at a given constant positive end-expiratory pressure (PEEP) increases the ventilation in the dependent parts of the lung by recruiting atelectatic lung regions (reduction of the dark grey oval symbols). Overdistension occurs in the non-dependent regions (increasing number of red circles). On the right, the decrease in compliance in the non-dependent ROI and its increase in the dependent ROI is explained in a P-V diagram. Additionally, the observed changes in the distribution of regional ΔC is shown. Middle panel: The effect of acute lung injury (ALI) with an increase in atelectatic lung (higher number of dark grey oval symbols) and the decrease in regional compliance is shown. Lower panel: Applying high levels of PEEP after ALI results in reduction of atelectasis (reduction of the number of dark grey symbols) along with an increase in compliance in the dependent ROI but also leads to a higher degree of overdistension in the non-dependent ROI (higher number of large red circles).
(TIF)

Figure S2 Low-flow inflation maneuver. Low-flow inflation manoeuver (pressure-volume (PV) maneuver) with the lower inflection point (LIP) identified on the inflation limb of the curve at the airway pressure of 20 cmH_2_O. Original tracing obtained in one of the studied animals (animal 7). The values of inhaled air volume and airway pressure by the end of inflation are indicated in the grey boxes. Paw, pressure at the airway opening, V_T_, tidal volume.
(TIF)

Text S1 Hypothesis.
(DOC)
