# Supplementary figures and images for: Effect of PEEP and Tidal Volume on Ventilation Distribution and End-Expiratory Lung Volume: A Prospective Experimental Animal and Pilot Clinical Study
Source: PLoS One. 2013 Aug 22;8(8):e72675. doi: 10.1371/journal.pone.0072675 (PMC3750001; doi:10.1371/journal.pone.0072675)

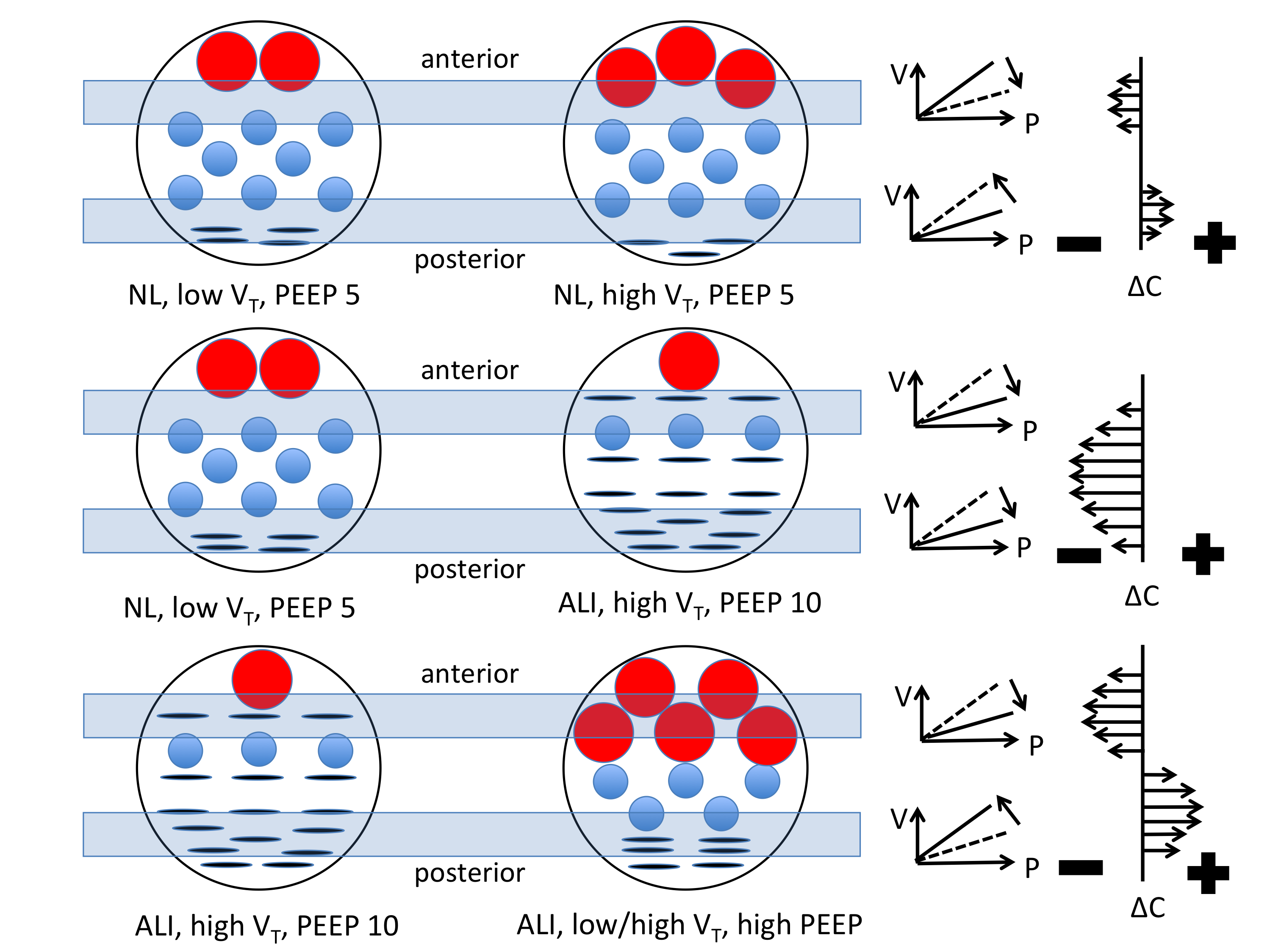

Supplement: Figure S1 — Explanation of the model. Schematic presentation of postulated changes in regional lung ventilation and regional respiratory system mechanics during different phases of the study protocol. Each large circle symbolizes ventilated lung volume. The small blue and large red circles represent normally aerated and overdistended lung regions, respectively. The oval dark grey symbols indicate atelectatic lung regions. The transparent grey bars show schematically two of the 32 regions of interest (ROI) used in our EIT analysis. (The sizes of these representative ROIs were enlarged to enable better visual perception.) The effect of an intervention is displayed from left to right showing the compliance change in the pressure (P)-volume (V) coordinates in the respective ROI and the assumed differences in regional compliance (ΔC) in the whole lung. Upper panel: An increase in tidal volume (VT) at a given constant positive end-expiratory pressure (PEEP) increases the ventilation in the dependent parts of the lung by recruiting atelectatic lung regions (reduction of the dark grey oval symbols). Overdistension occurs in the non-dependent regions (increasing number of red circles). On the right, the decrease in compliance in the non-dependent ROI and its increase in the dependent ROI is explained in a P-V diagram. Additionally, the observed changes in the distribution of regional ΔC is shown. Middle panel: The effect of acute lung injury (ALI) with an increase in atelectatic lung (higher number of dark grey oval symbols) and the decrease in regional compliance is shown. Lower panel: Applying high levels of PEEP after ALI results in reduction of atelectasis (reduction of the number of dark grey symbols) along with an increase in compliance in the dependent ROI but also leads to a higher degree of overdistension in the non-dependent ROI (higher number of large red circles). (TIF) [file pone.0072675.s001.tif]

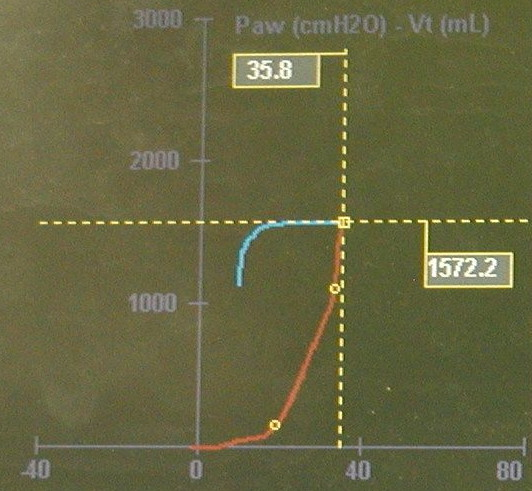

Supplement: Figure S2 — Low-flow inflation maneuver. Low-flow inflation manoeuver (pressure-volume (PV) maneuver) with the lower inflection point (LIP) identified on the inflation limb of the curve at the airway pressure of 20 cmH2O. Original tracing obtained in one of the studied animals (animal 7). The values of inhaled air volume and airway pressure by the end of inflation are indicated in the grey boxes. Paw, pressure at the airway opening, VT, tidal volume. (TIF) [file pone.0072675.s002.tif]
