# Supplementary material for: Effect of PEEP and Tidal Volume on Ventilation Distribution and End-Expiratory Lung Volume: A Prospective Experimental Animal and Pilot Clinical Study
Source: PLoS One. 2013 Aug 22;8(8):e72675. doi: 10.1371/journal.pone.0072675 (PMC3750001; doi:10.1371/journal.pone.0072675)
Supplement: Text S1 — Hypothesis. (DOC) [file pone.0072675.s003.doc]

**Effect of PEEP and tidal volume on ventilation distribution and end-expiratory lung volume: a prospective, randomized controlled crossover experimental animal study**

Günther Zick, Gunnar Elke, Tobias Becher, Dirk Schädler, Sven Pulletz, Sandra Freitag-Wolf, Norbert Weiler, Inéz Frerichs

**Text S1**

**Hypothesis**

In the supine, mechanically ventilated lung we assume different regions of aeration. Apart from normally aerated regions there exist atelectatic regions predominantly in the posterior, i.e. dependent parts of the lung and overinflated regions in the anterior, i.e. non-dependent parts. Since respiratory system compliance (C_RS_) is dependent on the ventilated volume C_RS_ is the highest in the normally ventilated lung regions whereas in the atelectatic as well as in the overinflated regions the compliance is lower. With increasing positive end-expiratory pressure (PEEP), recruitment of atelectatic regions is possible (PEEP recruitment) meaning that C_RS_ in the recruited regions will increase. At the same time, a certain amount of overinflation may occur in the anterior lung regions depending on the chosen tidal volume (V_T_). Recruitment potential is present if lung regions, which are atelectatic at the applied PEEP level open during inflation (tidal recruitment). To assess recruitment potential two different tidal volumes (low and high) may be applied and the change in regional C_RS_ calculated. If C_RS_ increases with the high V_T_ tidal recruitment can be assumed.

One could speculate that as long as tidal recruitment occurs at a given PEEP there should be potential for further recruitment. With increasing levels of PEEP one might thus be able to identify the threshold at which no longer tidal recruitment is present. Independent of this threshold, any increase in PEEP can lead to overinflation depending on the chosen V_T_. Here again the comparison of regional C_RS_ using different tidal volumes could guide the choice of the most adequate V_T_.

Figure S1 shows schematically the distribution of normal, atelectatic and overinflated lung regions along with regional changes in respiratory system compliance explaining the interpretation of the findings in different phases of our study.
